# Modified Fish Gelatin as an Alternative to Mammalian Gelatin in Modern Food Technologies

**DOI:** 10.3390/polym12123051

**Published:** 2020-12-19

**Authors:** Svetlana R. Derkach, Nikolay G. Voron’ko, Yuliya A. Kuchina, Daria S. Kolotova

**Affiliations:** Department of Chemistry, Murmansk State Technical University, 183010 Murmansk, Russia; derkachsr@mstu.edu.ru (S.R.D.); kuchinayua@mstu.edu.ru (Y.A.K.); kolotovads@mstu.edu.ru (D.S.K.)

**Keywords:** fish gelatin, amino-acid composition, sole-gel transition, rheology

## Abstract

This review considers the main properties of fish gelatin that determine its use in food technologies. A comparative analysis of the amino acid composition of gelatin from cold-water and warm-water fish species, in comparison with gelatin from mammals, which is traditionally used in the food industry, is presented. Fish gelatin is characterized by a reduced content of proline and hydroxyproline which are responsible for the formation of collagen-like triple helices. For this reason, fish gelatin gels are less durable and have lower gelation and melting temperatures than mammalian gelatin. These properties impose significant restrictions on the use of fish gelatin in the technology of gelled food as an alternative to porcine and bovine gelatin. This problem can be solved by modifying the functional characteristics of fish gelatin by adding natural ionic polysaccharides, which, under certain conditions, are capable of forming polyelectrolyte complexes with gelatin, creating additional nodes in the spatial network of the gel.

## 1. Introduction

Gelatin from bone and connective tissue of pigs and cattle is traditionally used in the food industry as a gelling agent [1,2,3,4]. However, the consumption of gelatin from these mammalian species contradicts ethnocultural and religious norms of a number of religions, and is also associated with the risk of contracting prion diseases (in particular, spongiform encephalopathy) [2,5,6,7,8]. In this regard, it seems relevant to look for alternative sources of food gelatin [5,9]. Such a source may be the connective tissue of fish [2,10,11,12,13], the industrial processing of which partially solves the problem of disposal and integrated use of waste from the fish processing industry [8,14,15,16,17]. The total world volume of fishing in 2018 is estimated at 179 million tons, and this figure is increasing every year [18]. According to various estimates, waste from fish processing can be up to85% of the total catch [14,18,19]. A significant percentage of waste (about 30%) is skin, bones, and scales with a high collagen content [8,14,16,18]. Therefore, the production and use of fish gelatin as a food structure-forming agent seems to be very promising. Currently, only 1.5% of gelatin is produced from fish collagen-containing raw materials, while 41% is produced from pig skin, 28.5% from bovine hides, and 29.5% from bovine bones [20].

Fish gelatin is characterized by a lower content of proline and hydroxyproline compared to gelatin from mammals [1,2,21,22]. This is especially true for gelatin obtained from cold-water species [1,23]. This leads to a deterioration in the gelling ability of fish gelatin, a decrease in the gelation and melting temperatures [21,24], a decrease in the gel strength [24,25], and increased consumption of gelatin as a food component for hydrogel formation [7,26]. Many studies have been devoted to finding ways to eliminate these serious disadvantages by treating fish gelatin with various physical and chemical cross-linkers: irradiation in various frequency ranges [7,27,28,29], high pressure [28,30], enzymatic modification [7,31], additions of mono- and disaccharides [7,32] and ferulic and caffeic acids [33,34]. However, the most effective and common way to improve gelling ability and rheological characteristics are the modification of fish gelatin with natural polysaccharides, for example, κ-carrageenan [6,35,36], sodium alginate [37,38,39], chitosan [29,40], gellan [6,36], gum arabic [41,42], and pectin [20,43]. This method of improving functional properties can increase the nutritional value of gelatin gels [29,39,43].

This review analyses the composition, structure, and functional properties of fish gelatin as an alternative to mammalian gelatin when used as a food gelling agent. The mechanism of modifying the gel-forming properties of fish gelatin and the rheological properties of gelatin gel by adding natural ionic polysaccharides is considered.

## 2. Amino-Acid Composition of Fish Gelatin

The amino acid sequence of a gelatin macromolecule can be described by the general formula Gly-X-Y, where the X position usually occupied by proline and Y position by hydroxyproline [1,7,9,19]. The amino acid triads Gly-Pro-Y, Gly-X-Hyp, and Gly-Pro-Hyp in macromolecular chains play a major role in the formation of triple collagen-like helices [19,44,45]. The comparative amino acid composition of gelatin obtained from various sources is presented in Table 1. Compared with gelatin from mammals, fish gelatin is characterized by a lower content of proline and hydroxyproline. At the same time, in terms of the content of these amino acids, gelatin from the skin of warm-water fish species (tilapia, tuna, black carp) is similar to gelatin from pork and calf skin.

The low content of proline and hydroxyproline, which are involved in the stabilization of collagen-like triple helices, leads to the fact that the secondary structure of fish gelatin is represented more by β-turn/β-shift structures than triple helices, as was found by FTIR spectroscopy [23]. This distinguishes it from mammalian gelatin [5,48,49]. In addition, [2] reported a negative impact of a large number of β-structures on the functional properties of fish gelatin.

## 3. Sole-Gel Transition and Rheological Properties of Fish Gelatin Gel

The amino acid composition, and hence the structural features, of fish gelatin is the reason that fish gelatin as a food gelling agent is inferior to mammalian gelatin in some functional characteristics (gelation and melting temperatures, rheological characteristics of solutions and gels) [1,2,42].

It was established by optical rotation that the conformational transition random coil → helix ends when the solution is cooled to a temperature of about 35 °C for gelatin from mammals [50], and to a temperature of 15 to 20 °C for gelatin from various cold-water fish species [1,21]. Below the conformational transition temperature, the triple collagen-like helices of gelatin are combined into a spatial network, forming a thermo-reversible viscoelastic hydrogel [44,45,51].

Table 2 shows the gelation and melting temperatures of 10% (*w/v*) gelatin gels from cold and warm water fish. For comparison, data for mammalian gelatin (porcine and bovine) are shown. Data is obtained from various literature sources.

As seen in Table 2, fish gelatin gels generally form and melt at a lower temperature than mammalian gelatin gels. At the same time, the values of the temperatures of gelation and the melting of gelatin gels from warm-water fish approximately correspond to the temperatures of gelatin from mammals. Thus, the melting point of gelatin gel from Black Tilapia leather is 28.9 °C [34]. There is a correlation between the content of proline and hydroxyproline (see Table 1) in gelatin and the temperatures of gelation and the melting of gels (see Table 2), which is also noted by Karim [2] and Wasswa [14].

Another significant limitation to the use of fish gelatin as a gelling agent in food technologies is the low rheological parameters (strength, elastic moduli) of gels as compared to mammalian gelatin [1,42,53]. This is also due to the reduced content of the amino acids, proline and hydroxyproline. Traditionally, the standard Bloom method is used to assess the strength of 6.67% (*w/v*) gelatin gels held at 10 °C for 17 h [1,42]. The Bloom strength of gelatin gels for cold-water species does not usually exceed 100 g; for warm-water species this is—200 g, while for gelatin from mammals it reaches 320 g and higher [25,42,53]. At the same time, the Bloom strength of gelatin gels for some species of warm-water fish (Nile tilapia, catfish, grass carp) is comparable and may even exceed the strength of mammalian gelatin gels [2,25].

Solution viscosity is the second most important commercial property of gelatin [25,45]. The viscosity of a gelatin solution is measured by a standard method at a concentration of 6.67% (*w/v*) and a temperature of 60 °C [1,34,53]. Commercial gelatin solutions have a viscosity of 2.0 to 7.0 MPas for most types, and over 13.0 Mpas for specialized types [34,45]. The viscosity of gelatin obtained from different fish species has different values, which depend on the specific characteristics of the fish raw materials, production conditions, molecular weight, and degree of polydispersity [14,34,53]. Thus, the viscosity of gelatin from the skin and bones of African catfish was 1.13 and 0.66 MPas, respectively; the viscosity of gelatin from the rainbow trout leather was 3.53 MPas; the viscosity of gelatin from the skin and bones of tiger-toothed croaker was 10.5 and 8.3 MPa, while the viscosity of farmed giant catfish skin gelatin is 112.5 MPas [34,53].

It should be noted that gelatin, which forms the least stable gel at 10 °C, is, at the same time, characterized by the highest values of solution viscosity at 60 °C in the region of about pI of gelatin [25]. For example, the viscosity of Alaska Pollock skin gelatin (cold water type) is 120 MPa (with a Bloom gel strength of 98 g), and the viscosity of tilapia skin gelatin (warm water type) is 38 MPas (Bloom 273 g) [14,34]. For comparison, the viscosity of pork skin gelatin is 47 MPas (Bloom 240 g) [14], while the viscosity of bovine gelatin is only 5.5 MPas (Bloom 323 g) [25].

Bloom gel strength and gelatin solution viscosity are spot measurements [1,45]. More complete information on the kinetics of changes in the viscoelastic characteristics of gelatin during gelation can be obtained by periodic oscillations [52]. The gelation process is kinetic because the storage modulus G′ can increase almost infinitely over time, although the most dramatic increase is observed in the first two hours [1,54,55]. It was shown by Yoshimura [24] that during cooling of a gelatin solution of 3.0% (*w/v*), the elastic modulus G′ began to increase sharply (the beginning of gelation) upon reaching a temperature of ~30 °C for pig gelatin, and only upon reaching ~21 °C for shark gelatin. Accordingly, at the same temperature values, a sharp drop in the tangent of the angle of mechanical losses, tanδ, was observed. Besides, when the gels were kept for 4 h at 4 °C, G′ reached ~2000 Pa for pig gelatin and was almost twice that for shark gelatin. When comparing the G′ values of gelatin gels obtained from cold-water fish and mammals, a much more significant difference is observed. Thus, the G′ of a bovine gelatin gel practically does not differ from the G′ of tilapia gelatin (warm-water species) [56], but exceeds the G′ of cod gelatin by about 10 times in a wide concentration range [26]. It was also noted by Haug [21] that gelatin gels from cold-water fish species (cod, haddock, pollack) have significantly lower G′ values than gelatin gels from cattle hide.

It is well known that the physical and functional properties of gelatin depend not only on their amino acid composition but also on their Bloom index [57,58,59], molecular weight distribution, on the relative contents of α-, β-, γ-chains and on the presence of protein fragments of low molecular weight [1,44,53]. So the viscosity of solutions [25] and strength of gels [1] are partially controlled by molecular weight and polydispersity of gelatin regardless of their source of origin. The fish gelatin extracted under mild conditions (pH5) showed higher content of α-chains, lower content of low molecular components and narrower molecular weight distribution then gelatin obtained under more aggressive acidic and alkaline conditions. This was found for gelatin from the skin of cold water species: Atlantic cod [23] and Atlantic salmon [60,61,62]. At the same time, salmon gelatin showed lower molecular weight compared with bovine gelatin [60]. Accordingly, fish gelatin samples extracted under pH5 with higher content of α-chains formed gels with higher values of gel strength, viscoelastic properties, gelling and melting temperatures [23,61,62].

## 4. Improving the Functional Properties of Fish Gelatin Food Gel

The noted serious disadvantages of fish gelatin in comparison with gelatin from mammals (see paragraph 3) can be eliminated by using natural polysaccharides that have the status of food additives [20,63]. Macromolecules of gelatin carry charged basic and acidic groups, as well as hydroxyl groups and hydrophobic hydrocarbon radicals (see Table 1). Therefore, gelatin can form polyelectrolyte complexes with ionic polysaccharides, entering with them into intermolecular electrostatic interactions [63,64,65]. Hydrogen bonds [66,67] and hydrophobic interactions [63,67] also play an important role in the stabilization of the polysaccharide–gelatin complexes.

The complexation of fish gelatin with polysaccharides depends on pH, as shown by the example of gelatin from cold-water fish with the anionic polysaccharide gum arabic in a wide pH range [41]. Since gelatin was extracted under alkaline conditions, its isoelectric point pI was 4.8. Concentrated emulsions, stabilized by gum arabic–fish gelatin complexes, with high values of the storage modulus G′ and low values of the loss modulus G″, are formed in the acidic pH range (3.6) in the region below the pI of gelatin. Obviously, under these conditions (provided that the dissociation of the carboxyl groups of gelatins is suppressed), the electrostatic interactions of negatively charged gum arabic with basic gelatin residues are most effective. Measurement of the turbidity of the solution shows that, with a further decrease in pH, large insoluble aggregates of gum arabic with fish gelatin are formed [42]. A similar effect of an increase in the G′ of emulsions stabilized with fish gelatin, upon the addition of another anionic polysaccharide, pectin, has been considered by [43].

**Figure 1:**
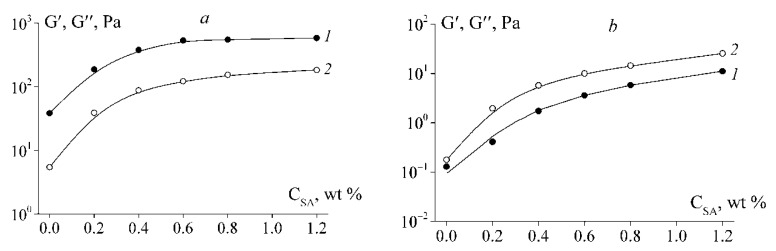
Dependencies of storage G′ (1) and loss G″ (2) moduli on the concentration of sodium alginate C_SA_ in fish gelatin gels at 4 °C (**a**) and 14 °C (**b**). Gelatin concentration is 10 wt%.

The anionic marine polysaccharide proposed to improve the functional properties of fish gelatin, in order to replace mammalian gelatin in food technologies; is sodium alginate [37,38,39]. At low pH values of 3.5 (below pI gelatin), mixtures of sodium alginate and gelatin from the skin of cold-water fish species (cod, pollock, haddock) exhibit a high foaming ability and form stable food foams [39]. This is due to the favourable conditions for electrostatic interactions of gelatin with alginate in this pH range. In the pH region below pI, fish gelatin forms large insoluble aggregates with sodium alginate [37]. The addition of the anionic polysaccharide sodium alginate leads to an increase in the elastic moduli (storage modulus G′ and loss modulus G″) of fish gelatin gel [68] (Figure 1).

This is in agreement with the results of studying the interactions of anionic gum arabic with fish gelatin described by Anvari [41], and Chung [42].

Sow [38] proposed a schematic model of the mechanism for the formation of the spatial network of the complex gel of sodium alginate with fish gelatin from tilapia, due to electrostatic interactions between biopolymers. The area of ratios between polysaccharide and gelatin, in which the most durable gels were formed, was established.

The melting point and elastic modulus of gelatin gel from the skin of cold-water fish species increases with the addition of κ-carrageenan [35]. This is due to the formation of a complex κ-carrageenan–fish gelatin network, in which electrostatic interactions between negative κ-carrageenan groups and positive gelatin groups create additional nodes. A similar effect of κ-carrageenan additives on the melting point and strength characteristics of gelatin gel from tilapia fish skin has been described by Pranoto [36]. Another anionic polysaccharide, gellan, has a similar effect [36]. The strengthening effect of charged polysaccharides is primarily due to the presence of electrostatic interactions. In this case, hydrogen bonds play a significant role in the complexation of κ-carrageenan and gellan with fish gelatin during the modification of the gel nanostructure [6]. This is in agreement with the conclusions obtained when describing the mechanism of the complexation of κ-carrageenan with mammalian gelatin [64,67].

**Figure 2:**
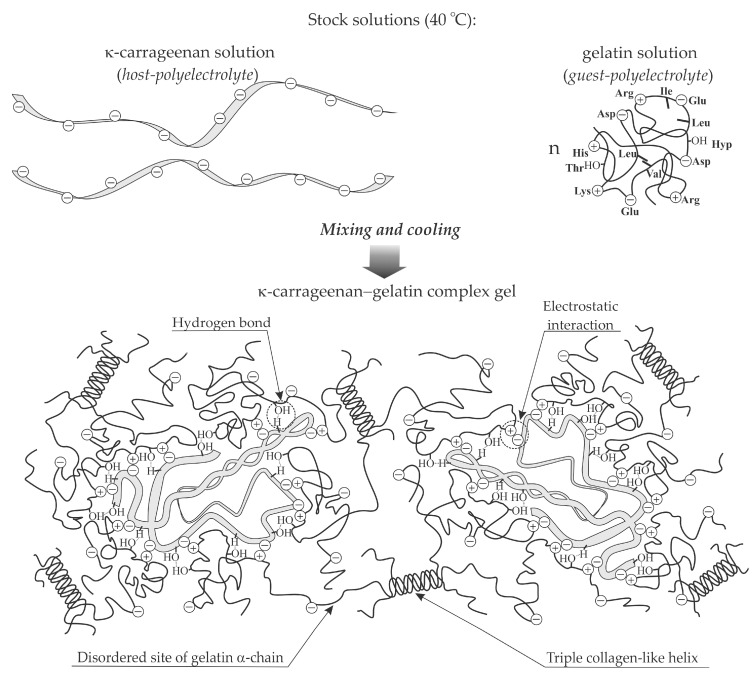
Schematic model of the formation of a complex κ-carrageenan–gelatin gel network.

The schematic model of the formation of a complex κ-carrageenan–gelatin gel network is presented in Figure 2. Using high resolution ^1^H NMR spectroscopy it was shown that, upon cooling, electrostatic interactions and hydrogen bonds between κ-carrageenan and gelatin start to dominate, while the role of hydrophobic interactions minimizes [67]. The contribution of electrostatic interactions and hydrogen bonds in κ-carrageenan–gelatin complexation was also indicated by FTIR spectroscopy [64]. Complexes aggregation occurs due to the formation of intermolecular triple collagen-like helices of gelatin α-chains [64,67]. In this case, as is well known, hydrogen-bonded water network makes a major contribution to the stabilization of the collagen-like helices, increasing their crystallinity [44,69,70]. In particular, such a stabilizing role of water has been noted for gelatin from the skin of Atlantic salmon [60,61].

In general, it should be noted [6,35,36] that fish gelatin modified with anionic polysaccharides from seaweed κ-carrageenan and gellan can be a good alternative to mammalian gelatin (porcine or bovine) in the food industry.

Unlike the anionic polysaccharides discussed above, the cationic polysaccharide chitosan can be effectively used to improve the functional properties of fish gelatin in the pH range above pI, where the electrostatic interactions of charged carboxyl groups of gelatin and positive groups of chitosan will be most pronounced [29]. However, it was found using the FTIR method that, in the acidic pH region (5) below pI of gelatin extracted under acidic conditions (8.9), where both biopolymers carry a positive charge, fish gelatin interacts with chitosan, mainly due to hydrogen bonds, but electrostatic interactions also make a small contribution [40]. A similar feature of the interaction of chitosan with gelatin from bovine skin at pH range of 3.2–3.9 below pI of gelatin extracted under alkaline conditions (4.7) was noted by Voron’ko [71].

## 5. Conclusions

Fish gelatins, in contrast to gelatin obtained from mammals, has a lower content of proline and hydroxyproline, amino acids responsible for the stabilization of collagen-like triple helices, and also of lower molecular weight. This is especially true for cold-water fish species and, respectively, for fish gelatin extracted under more aggressive conditions. In this regard, the secondary structure of fish gelatin, in contrast to mammalian gelatin, is represented more by β-turn/β-shift structures than triple helices. As a result, fish gelatin as a food gelling agent has several limitations compared to the traditionally used porcine or bovine gelatin. It has lower gelling and melting temperatures, reduced gel strength, and higher consumption in the production of structured food products.

Replacing porcine and bovine gelatin in the food industry with fish gelatin is very tempting because it will significantly expand the sales market by attracting potential consumers, for whom, at the moment, the consumption of products containing gelatin from the mentioned mammalian species is unacceptable for religious and ethnocultural reasons. Besides, the use of fish gelatin will eliminate the risk of infection by prion diseases and will partially solve the problem of disposal of waste from the fish processing industry. The disadvantages of fish gelatin as a food gelling agent can be eliminated by modifying its functional characteristics by the addition of natural ionic polysaccharides capable of forming polyelectrolyte complexes with gelatin. The formation of complexes in a pH range below pI of gelatin (for anionic polysaccharides) and above pI of gelatin (for cationic polysaccharides), due to electrostatic interactions (mainly) and hydrogen bonds, will lead to an increase in the temperatures for formation and melting of gels, and a strengthening of the spatial network of the gelatin hydrogel.

## Figures and Tables

**Table 1:** Amino-acid composition of some fish gelatins compared to pork and calfskin gelatine.

Source	Cold Water Fish Skin			Warm Water Fish Skin			Pork Skin [2]	Calf Skin [21]
	Cod [10]	Hake [10]	Alaska Pollock [2]	Tilapia [1,2]	Tuna [46]	Black Carp [19,47]		
Amino Acid Composition (Residues Per 1000 Total Amino Acid Residues)								
Glycine (Gly)	344	331	358	347	336	314	330	313
Basic groups	99	97	91	86	90	88	86	101
Lysine (Lys)	29	28	26	25	25	29	27	34
Hydroxylysine(Hyl)	6	5	6	8	6	2	6	11
Histidine (His)	8	10	8	6	7	4	4	5
Arginine (Arg)	56	54	51	47	52	53	49	51
Carboxylic groups	130	123	125	117	115	126	118	116
Aspartic acid (Asp)	52	49	51	48	44	48	46	45
Glutamic acid (Glu)	78	74	74	69	71	78	72	71
Hydroxylic groups	142	134	146	140	150	131	147	144
Serine (Ser)	64	49	63	35	48	37	35	37
Threonine (Thr)	25	22	25	24	21	25	18	18
Hydroxyproline (Hyp)	50	59	55	79	78	69	91	86
Tyrosine (Tyr)	3	4	3	2	3	0	3	3
Hydrophobic groups	286	314	280	309	321	336	322	326
Alanine (Ala)	96	119	108	122	119	119	112	114
Valine (Val)	18	19	18	15	28	22	26	22
Leucine (Leu)	22	23	20	23	21	22	24	25
Isoleucine (Ile)	11	9	11	8	7	12	10	11
Proline (Pro)	106	114	95	119	117	133	132	135
Phenylalanine (Phe)	16	15	12	13	13	14	14	13
Methionine (Met)	17	15	16	9	16	14	4	6

**Table 2:** Gelling and melting temperatures of cold and warm water fish gelatin gels compared to mammalian gelatin gels for 6.67–10% (*w/v*) gel.

Gelatin	Gelling Temperature, °C	Melting Temperature, °C	References
Cold water fish gelatin	4–8	16–18	[1]
	7–11	11–19	[2]
	4–5	12–13	[5]
	4–12	<17	[9]
	4–10	13–16	[21]
		16–21	[34]
	7–9	18–20	[52]
	12	14–21	[53]
Warm water fish gelatin	21–22	28–29	[1]
	15–20	20–27	[2]
	18–19	24–29	[9]
		22	[24]
		22–29	[34]
	19–22	24–25	[53]
Mammalian gelatin	26–27	33–34	[1]
	20–25	28–31	[2,34]
		29	[24]
